# The effectiveness of solar disinfection water treatment method for reducing childhood diarrhea: a systematic review and meta-analysis protocol

**DOI:** 10.1186/s13643-020-01288-8

**Published:** 2020-02-12

**Authors:** Negasa Eshete Soboksa, Sirak Robele Gari, Abebe Beyene Hailu, Dereje Oljira Donacho, Bezatu Mengistie Alemu

**Affiliations:** 1 Ethiopian Institute of Water Resources, Addis Ababa University, Addis Ababa, Ethiopia; 2 Department of Environmental Health sciences, Jimma University, Jimma, Ethiopia; 3 College of Health and Medical Sciences, Haramaya University, Harar, Ethiopia

**Keywords:** Solar disinfection, Water treatment, Childhood diarrhea

## Abstract

**Background:**

Several studies employing the effectiveness of solar disinfection water treatment method for reducing diarrhea have reported heterogeneous outcomes, necessitating a systematic review to provide an exhaustive summary of current evidence. Thus, the objective of this review is to pool out the available evidence on the effectiveness of solar disinfection water treatment method for reducing childhood diarrhea.

**Methods:**

Searches will be conducted in PubMed/Medline, Scopus, Google Scholar, Cochrane Library databases, and reference of other studies published through in December 2019. Studies that compare the diarrhea incidence among the intervention group who were exposed to solar disinfection water treatment and the control group who were not exposed to such water treatment were included. The primary outcome of the study is the change in observed diarrhea incidence risk from baseline to post-intervention. Randomized controlled trial study designs will be included. Selected studies will be critically appraised by two independent reviewers. Extracted data will include details about the interventions, populations, study methods, and outcomes of significance to the review question and objectives. Effect sizes will be expressed as risk ratio, and their 95% confidence intervals will be calculated for analysis.

**Discussion:**

This review and meta-analysis will systematically explore and integrate the evidence available on the effectiveness of solar disinfection water treatment method for reducing diarrhea. In this review, information about the potential impact of solar disinfection water treatment to inactivate pathogenic microbes for reducing diarrhea will be gathered and summarized. The findings from this study will provide directions for future research and public health professionals with an understanding of the importance of solar disinfection water treatment and point to directions for applicability of the interventions in the community.

## Background

Diarrhea is the second leading cause of death in children under 5 years old, and estimated 2.5 billion cases of diarrhea occur and also responsible for killing around 760,000 children every year. More than half of these cases are in Africa and South Asia, where the attacks of the diseases are more likely to result in death or other severe outcomes [1]. This diarrhea related to death in developing countries is mostly attributable to inadequate water, sanitation, and hygiene [2]. The deaths of 297,000 children aged under 5 each year could be avoided if the risk factors were addressed [3]. Previous systematic review findings suggest that point-of-use water treatment is one of the most effective strategies to reduce this disease [4–7].

The importance of household water treatment and safe storage (HWTS) in the reducing of diarrheal disease has been increasingly recognized [8]. Solar disinfection (SODIS) method is recognized as one viable HWTS option [9]. It is the simplest, cheapest technologies and effective water treatment method that is applicable to emergencies, especially when no chemical disinfectants are available [10]. Solar water disinfection is one of the proven and field-tested household water treatment options that are currently being promoted by many organizations [1, 9]. The method relies on disposable transparent plastic or glass containers which are then exposed to the sun and its germicidal effect is based on the combined effect of thermal heating of solar light and UV radiation [11–13]. Since SODIS is simple to use and inexpensive, the method has spread throughout the developing world and is in daily use in more than 50 countries in Asia, Latin America, and Africa. More than 5 million people disinfect their drinking water with the SODIS technique as the report of a systematic review [13].

Solar disinfection has been repeatedly shown to be effective for eliminating microbial pathogens and reduce diarrheal morbidity, but its effectiveness is limited to waters of low turbidity [11, 14]. Previously done cluster-randomized controlled trial studies showed that solar disinfection of drinking water reduces diarrhea among under-five children [15–17]. A matched case-control study done in India also concludes that solar disinfection of water can significantly decrease diarrhea morbidity in children [18]. Other study findings reported from Kenya show that children drinking solar disinfected water had a significantly lower risk of severe diarrheal disease over 8705 two-weekly follow-up visits; 2-week period prevalence was 48.8% compared with 58.1% in controls, corresponding to an attributable fraction of 16.0% [17].

Previously conducted studies show that solar disinfection is highly important among household water treatment and safe storage for the reduction of diarrhea. But, the reported findings showed that heterogeneous outcomes and a systematic review and meta-analysis have not been done to compare the evidence of the relative effectiveness of solar disinfection water treatment method for reducing diarrhea. Therefore, the objective of this review is to pool out the available evidence on the effectiveness of solar disinfection water treatment method for reducing diarrhea. The research question of this review is “Does solar disinfection water treatment method improves the microbial quality of water and reduce diarrhea?”

## Methods

### Study design and protocol

This protocol follows the Preferred Reporting Items for Systematic Review and Meta-Analysis Protocols (PRISMA-P) statements [19] (see Additional file 1).

### Criteria for considering studies for this review

Studies that meet the following criteria will be included in this review.

### Types of studies

Only randomized controlled trial studies done to assess the effectiveness of solar disinfection water treatment method for reducing diarrhea will be included in this review.

### Participants

The review will include all children who live everywhere in the world regardless of sex, ethnicity, and socioeconomic status.

### Interventions and comparator

This review will consider studies that will evaluate the effectiveness of solar disinfection as a water treatment method for the reduction of diarrhea. Studies that compare the diarrhea incidence among the intervention group children who were exposed to solar disinfection water treatment and the control group children who were not exposed to such water treatment will be considered.

### Types of outcome measures

This review will consider studies that include incidence rate of diarrhea (non-bloody, with dehydration and dysentery), defined as number of diarrhea (three or more loose or watery stools during a period of 24 h or any loose stool which contained blood or mucus) episodes per child per year obtained from a daily assessment of the individual diarrhea occurrence. The primary outcome of this study will be the change observed in diarrhea incidence after solar disinfection was applied for the treatment of drinking water.

### Information sources and search strategy

An electronic database search will be carried out to identify appropriate peer-reviewed articles that meet the inclusion criteria. We will search PubMed/Medline, Scopus, Google Scholar, Cochrane Library databases, and reference of other studies. The search of the literature will be conducted in December 2019. The search strategy will be limited to studies published in the English language literature. We will use the following keywords terms (also in combination with MESH terms): (solar energy OR sunlight) AND (water disinfection OR water purification OR water treatment) AND (diarrhea OR diarrhoea OR dysentery) AND (child OR children OR childhood). A full search strategy for PubMed/Medline and Cochrane databases is detailed in Table 1. The results of the search and the full process for selecting included studies will be reported in full in the final report and presented in a PRISMA flow diagram [20].

**Table 1:** Search strategy for Pubmed/Medline databases

S/no.	Query
1.	Child [tw] OR childhood [tw] OR children [tw]
2.	“child”[MeSH]
3.	“sunlight”[MeSH] OR “solar energy”[MeSH]
4.	solar energy [tw] OR sunlight [tw]
5.	“water purification”[MeSH]
6.	water purification [tw] OR water treatment [tw]
7.	“Diarrhea/prevention and control”[MeSH] OR “dysentery/ prevention and control ”[MeSH]
8.	diarrhea [tw] OR diarrhoea [tw] OR dysentery [tw]
9.	1 OR 2
10.	3 OR 4
11.	5 OR 6
12.	7 OR 8
13.	9 AND 10 AND 11 AND 12
14.	Limit 13 to RCT AND English

### Data collection and analysis

#### Data management and selection of studies

The searched results will be managed using the Mendeley Desktop reference management software version 1.19.5 (Mendeley Ltd., Elsevier, Netherlands). The screening of studies will be conducted by two independent review authors (NES and DOD). The articles found by searches in databases will be evaluated for inclusion at three levels, i.e., by title, then by abstract, and finally by the full text. The full text of selected studies will be retrieved and assessed in detail against the inclusion criteria. Discrepancies will be discussed between reviewers and refine inclusion criteria. For the screening of articles at full text level, rejection of an article will be decided by the review team upon suggestion of the first reader. Details regarding the final decision of inclusion of articles will be clarified and archived in a database. In cases of uncertainty in the decision to include or exclude an article, the reviewer will include this article for the next level of screening. The documents without abstracts will be screened at the full text level. A list of articles excluded at full text level will be provided in the systematic review and accompanied by reasons for exclusion.

#### Data extraction

Data will be extracted from studies included in the review using a prepared data extraction tool form by two independent review authors (NES and DOD). For each study, authors’ name, place and year of publication, data on sample size and characteristics, characteristics of interventions performed, instruments used to assess outcomes, results of included studies, and follow-up of the study will also be extracted. The data extraction form will be pretested with three randomized controlled trials similar to those eligible in this review. Any disagreements that arise between the reviewers will be resolved through discussion or with a third reviewer (BMA).

#### Risk of bias of included studies

The methodological quality of the studies that meet the selection criteria will be evaluated independently by two authors (NES and DOD) using revised Cochrane risk-of-bias tool for randomized trials outlined in the Cochrane Handbook for Systematic Reviews of Interventions [21]. The tool is structured into five domains through which a bias might be introduced into the result. These were identified based on both empirical evidence and theoretical considerations. The rating for each bias criteria of the two authors will then compare. Disagreements between the two authors on individual bias criteria will be identified and discussed in an attempt to reach a consensus. Any disagreements that arise between the reviewers will be resolved through discussion or with a third reviewer.

#### Measures of treatment effect

It is expected that SODIS water treatment intervention reduces the relative incidence of diarrhea and we will use the relative risk (RR). RR will be estimated by using the following data: the number of participants who is not exposed to diarrhea and the total number of participants in each group. RR of more than one indicated that solar disinfection water treatment result in a greater chance of decreasing diarrhea.

#### Dealing with missing data

We will contact the authors for missing data and clarity of primary studies if required; such inclusions will be reported in the review.

#### Data synthesis

We will conduct a narrative synthesis first to describe study details, participant and intervention characteristics, and outcomes of the included studies. Then, meta-analysis will be conducted using random effects and fixed effects when at least three studies are comparable in design and protocol using the Comprehensive Meta-Analysis version 3.3 (Biostat, USA). We will calculate 95% confidence intervals and two-sided *p* values for the outcome. We will perform subgroup analyses according to the length of follow-up and based on types of interventions used (SODIS only and SODIS plus another methods used, e.g., turbidity reduction).

#### Assessment of heterogeneity

Heterogeneity will be assessed statistically using chi-square test (Q-test) statistics and inverse variance index (*I*^2^). *I*^2^ values will be classified as follows: no relevant heterogeneity (0–25%), moderate heterogeneity (25–50%), and significant heterogeneity (>  50%) [22]. Forest plots will be generated to present the pooled estimates where there are two or more RCTs of sufficient trials and statistical data. Funnel plots of the trial’s RR will be evaluated for publication bias. Sensitivity analyses will be repeated after exclusion of studies with a high risk of bias. The presence of publication bias will be examined using a funnel plot and Egger’s test. Sensitivity analyses will be repeated 10 times after excluding one study to observe the impact of individual study on the overall risk ratio.

#### Assessment of the quality of the evidence

The Grading of Recommendations, Assessment, Development, and Evaluation (GRADE) approach for grading the certainty of evidence will be followed by inserting appropriate citation, and a summary of findings will be created using GRADEPro GDT version 3.6.1 /2019 (McMaster University, ON, Canada) [23, 24]. According to GRADE, evidence quality assessment is performed for each outcome, and the combined available evidence is considered. The GRADE approach will classify the quality of the evidence into four levels: high, moderate, low, and very low based on the comprehensive assessment of inconsistency, indirect evidence (not generalizable), inaccuracy, and publication bias. These levels represent confidence in the estimation of the treatment effects presented. The level of evidence and strength of recommendation will be determined by discussion involving all authors.

## Discussion

This review and meta-analysis will systematically explore and integrate the evidence available on the effectiveness of solar disinfection water treatment method for reducing diarrhea. In this review, information about the potential impact of solar disinfection water treatment to inactivate pathogenic microbes for reducing diarrhea will be gathered and summarized. The review will be reported according to the PRISMA guidelines [20] and submitted to appropriate the journal for publication. The findings from this study will provide directions for future research and public health professionals with an understanding of the importance of solar disinfection water treatment and also point to directions for applying of the interventions in the community. The limitation of this review is that it will include only English language peer-reviewed studies.

## Supplementary information

**Additional file 1.** Filled PRISMA-P 2015 Checklist for the study on the title ‘the effectiveness of solar disinfection water treatment method for reducing diarrhea: A systematic review and meta-analysis protocol’ by Negasa et al.

## Data Availability

Not applicable

## Abbreviations

GRADE: Grading of Recommendations Assessment, Development, and Evaluation
HWTS: Household water treatment and safe storage
PRISMA-P: Preferred Reporting Items for Systematic review and Meta-Analysis Protocols
RCTs: Randomized controlled trials
SODIS: Solar disinfection
